# Preparation and Characterization of Zinc Hydroxystannate Coated by Aluminum Phosphate and Its Application in Poly(acrylonitrile-*co*-vinylidene chloride)

**DOI:** 10.3390/polym12061365

**Published:** 2020-06-17

**Authors:** Ji Eun Song, Ji Su Kim, Daeyoung Lim, Wonyoung Jeong

**Affiliations:** 1 Human Convergence Technology R&D Department, KITECH, Ansan 15588, Korea; mplala@kitech.re.kr (J.E.S.); nrhope2@kitech.re.kr (J.S.K.); zoro1967@kitech.re.kr (D.L.); 2 Department of Organic and Nano Engineering, Hanyang University, Seoul 04763, Korea

**Keywords:** flame retardant, zinc hydroxystannate, modacrylic fiber, aluminum phosphate, zinc hydroxystannate coating

## Abstract

In this study, zinc hydroxystannate ([ZnSn(OH)_6_], ZHS) was coated with aluminum phosphate (AlPO_4_, ALP) to prepare the ZHS-ALP composite. During the coating process, the reaction conditions, such as the ALP to ZHS molar ratio, were controlled, and the morphology of the products was characterized by scanning electron microscopy (SEM). The prepared composites were introduced into poly(acrylonitrile-*co*-vinylidene chloride) (PANVDC), and the change in compatibility between ZHS and the polymer matrix was characterized. The results showed that ALP-ZHS (1:1), which was prepared by ALP-ZHS composite molar ratio of 1:1, could improve the dispersion and compatibility of ZHS in the polymer matrix and decrease the hydrophilicity and viscosity. Moreover, the ALP-ZHS composite had a better flame-retardant effect on PANVDC than ZHS alone. PANVDC could pass the V-0 rating in UL94, particularly the highest limiting oxygen index (LOI) value of 33.2% obtained when the ALP-ZHS (1:1) composite was added to PANVDC.

## 1. Introduction

Halogenated flame retardants are used widely in reducing the fire hazards of polymeric materials. On the other hand, the halogen-containing flame retardants usually cause serious environmental contamination because they can produce poisonous substances during the combustion of halogen-containing composites [1]. Therefore, halogen-free and low smoke fire retardant composites have been applied in polymer matrices because of their desired environmental acceptance. Among them, inorganic compounds are considered the most promising ones because they produce low smoke and low toxicity during combustion [2]. Zinc hydroxystannate ([ZnSn(OH)_6_], ZHS), which is an inorganic tin-containing compound, has critical advantages over many other flame retardants, such as low addition and high efficiency. Hence, it has been used as an effective halogen-free flame retardant in a wide range of polymeric materials [3]. Nevertheless, there are some limits in the manipulation of ZHS, which tend to aggregate, resulting in their non-uniform dispersion and poor compatibility in a polymer matrix [4].

To deal with the above problems, coating the ZHS surface is effective in improving the particular properties of ZHS. Depending on the type of coating material, the water resistance and compatibility of ZHS in the polymer matrix can be enhanced [5].

In previous related research, ZHS coatings were conducted using hydrated inorganic fillers, such as aluminum hydroxide (ATH) and magnesium hydroxide (MH) [6]. On the other hand, there have been few studies on ZHS coatings with organic group composites. Organic compounds are effective in improving the dispersion and thermal stability of the inorganic flame retardant in the polymer matrix.

Aluminum phosphate (AlPO_4_, ALP), which is included in the organic group composite, is effective in improving the dispersion and thermal stability of the inorganic flame retardant in the polymer matrix [1]. Therefore, in this study, ALP was selected as the coating material for ZHS to enhance the compatibility and flame retardancy of ZHS [7].

The primary purpose of this study was to improve the dispersion and compatibility between ZHS and the polymer matrix but maintain the flame retardancy of ZHS. For this, ZHS was coated by synthesizing with ALP (hereinafter, ALP-ZHS composite) and introduced into poly(acrylonitrile-*co*-vinylidene chloride) (PANVDC). The PANVDC, which is composed by co-polymerized acrylonitrile and vinylidene chloride, is an inherently flame resistant. Although it burns when directly exposed to flame, it does not melt and is self-extinguishing when the flame is removed. The flame retardancy of PANVDC can be improved by flame-retardant additives [8,9]. For this, the ALP-ZHS composite was first prepared, and the coating condition, such as the molar ratio of ALP against ZHS, was controlled by measuring the surface properties, viscosity, and flame retardancy of ALP-ZHS composite-doped PANVDC.

After introducing the ZHS and ALP-ZHS composites in the PANVDC, the changes in chemical properties were also characterized by Fourier transform infrared (FTIR) spectroscopy, X-ray photoelectron spectroscopy (XPS), X-ray diffraction (XRD), and thermogravimetric analysis (TGA). The flame-retardant properties of the PANVDC polymer were evaluated by vertical burning (UL94), limiting oxygen index (LOI), and micro combustion calorimeter (MCC).

Therefore, this assessed the coating method of ZHS by ALP and evaluated the effects of the compatibility, thermal stability, combustion performance, and chemical properties in PANVDC.

## 2. Experimental

### 2.1. Materials

Zinc hydroxystannate (ZHS, ZnSn(OH)_6_) was purchased from Ditto Technology Co., Ltd. (Gunpo, Korea). The following chemicals were obtained from Sigma Aldrich (St. Louis, Mo, USA): sodium dodecyl sulfate (SDS), cetyltrimethylammonium bromide (CTAB), and aluminum hydroxide (Al(OH)_3_). PANVDC copolymer was a commercial flame retardant modacrylic with an AN to VDC. The weight average molecular weight of PANVDC was 170,000 Mw. Phosphoric acid (H_3_PO_4_) procured from Alfa Aesar Co., Inc. (Lancashire, UK) and dimethyl sulfoxide (DMSO) acquired from Samchun Chemical Co., Ltd. (Seoul, Korea).

### 2.2. Methods

#### 2.2.1. Preparation of ZHS Composites Coated by ALP (ALP-ZHS Composite)

In this paper, H_3_PO_4_ (98 g/mol) and Al(OH)_3_ (78 g/mol) were used as raw materials to prepare the aluminum phosphate (AlPO_4_, ALP). This study analyzed the reaction product and its polymerization extent under different coating conditions, such as the ALP molar ratio against ZHS.

The ZHS mixture was prepared by adding ZHS (10 g), SDS (0.02 g), and CTAB (0.2 g) to 200 mL of distilled water and dispersed ultra-sonication. After dispersion, aluminum hydroxide (Al(OH)_3_) was added to the ZHS mixture at Al(OH)_3_:ZHS molar ratio of 1:5, 1:1, 2:1 and phosphoric acid (H_3_PO_4_) was also added at a H_3_PO_4_: ZHS molar ratio of 1:5, 1:1, 2:1. Those samples are called ALP-ZHS(1:5), ALP-ZHS(1:1) and ALP-ZHS(2:1) composites. Each mixture was stirred at 50 °C for 1.5 h The ZHS composites coated by synthesizing ALP was centrifuged, washed several times with methanol, and dried overnight at 70 °C.

#### 2.2.2. Preparation of PANVDC Polymer-added ALP-ZHS Composite

The flame-retardant PANVDC samples were prepared as follows. PANVDC (8.7 g) was dissolved into DMSO solution (21.3 mL) in which ALP-ZHS composites were previously dispersed by ultra-sonication. ALP-ZHS composites were added by the amount 15% of the weight of PANVDC powder added. Depending on the ALP and ZHS molar ratio, the samples are called PANVDC/ALP-ZHS (1:5), PANVDC/ALP-ZHS (1:1), and PANVDC/ALP-ZHS (2:1), respectively. The dope was cast onto a glass substrate, pilled, and dried at 100 °C for 1 h to form a casting film.

#### 2.2.3. Characterization

The morphological structures of the ALP-ZHS composites were examined by scanning electron microscopy (SEM, JSM-6700F, JEOL Ltd., Tokyo, Japan). The composites were immersed in liquid nitrogen and fractured. The water contact angle (WCA) of the films composed by PANVDC, PANVDC-doped ZHS (hereinafter, PANVDC-ZHS), and PANVDC/ALP-ZHS were measured using a WCA measurement system (SEO, Phoenix-MT (T), Suwon, Korea) and its software (Surface Ware 9, Surface Electro Optics, Suwon, Korea) [10].

The viscosity of the PANVDC polymer solutions, including the ZHS and ALP-ZHS composites, was measured using a rotational rheometer with a small sample adapter on the Brookfield DV2T Viscometer (AMETEK. Inc., Mass., USA), respectively. The measurements were conducted at 50 °C under 10 rpm. The ratio of viscosity change (%) was calculated according to Equation (1).

(1) $$\mathrm{Ratio}\;\mathrm{of}\;\mathrm{viscosity}\;\mathrm{change}\;\left(\%\right)=\frac{V_{1}-V_{0}}{V_{1}}\times 100$$

where *V*_0_ is the viscosity of the polymer, and *V*_1_ is the viscosity of the polymer-added ZHS and ALP-ZHS composites, respectively.

The chemical structures of the samples were analyzed by Fourier transform infrared (FTIR, FT/IR-670Plus, Jasco International Co. Ltd., Tokyo, Japan) spectroscopy was performed between 4000 cm^−1^ and 450 cm^−1^ at a resolution of 4 cm^−1^. The samples were ground and mixed at 2% in potassium bromide (KBr). The baselines for each sample spectrum were normalized using spectrum software. The crystalline structures of the samples were characterized by powder XRD, D/MAX 2550V, D8 ADVANCE, BRUKER INC., Karlsruhe, Germany) using CuK radiation (λ = 1.5418 Å; scanning speed: 0.5/s.)

The vertical burning tests were conducted according to the UL94 (Underwriter’s Laboratory, thin material vertical burning test) under controlled laboratory conditions. The UL94 test is performed with a 20 mm vertical flame by twice contacting a molded sample with dimensions of 127 × 12.7 × thickness in mm for 10 s. To pass UL94 V2, the flame should extinguish within 30 s after each ignition. Burning drips are allowed. For UL94 V0 the flame should extinguish within 10 s after each ignition, with less than 50 s as total burn for 5 samples and no burning drips [11,12].

The LOI was tested according to ASTM D2863. The test specimens had dimensions of 100 mm × 6.5 mm × 3 mm [13,14]. Thermogravimetric analysis (TGA, TA Q500, TA Instruments, New Castle, DE, USA) was performed from room temperature to 800 °C at a heating rate of 20 °C/min in air [15].

A MCC test was conducted using a pyrolysis combustion flow calorimeter (Fire Testing Technology Ltd., East grinstead, UK), according to ASTM D 7309-19a [16]. Samples (5 mg) were exposed at a heating rate of 1 C/s and an O_2_ flow rate of 20 cc/min.

## 3. Results and Discussion

### 3.1. Preparation of ALP-ZHS Composite

**Figure 1:**
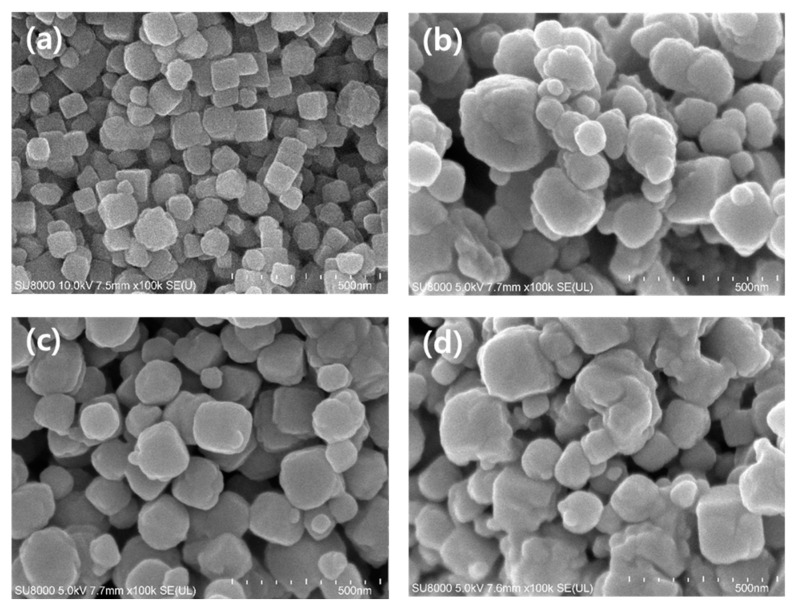
SEM images of (**a**) pure ZHS, (**b**) ALP-ZHS (1:5), (**c**) ALP-ZHS (1:1), and (**d**) ALP-ZHS (2:1) composites prepared at 50 °C for 1.5 h (magnification: ×100,000).

Figure 1 shows the surface morphology of pure ZHS and ALP-ZHS composites prepared by different molar ratios of ALP against ZHS (1:5, 1:1, and 2:1), respectively. The surface of pure ZHS Figure 1a had a regular square shape with sharp edges. After ZHS was coated with ALP, the ZHS particles became globular in shape with the coated-like surface, which was an accumulation of many small particles of ALP [5]. The ALP-ZHS (1:1) composite Figure 1c was more spherical with a well-dispersed morphology, a uniform coating, and a particle diameter of 150 nm to 160 nm. On the other hand, the ALP-ZHS (1:5) composite Figure 1b and ALP-ZHS (2:1) Figure 1d composites had irregularly coated particles, resulting in unstable and aggregated particles. In particular, the particle diameter of the ALP-ZHS (2:1) composite increased from around 80 nm (pure ZHS) to 225 nm. This might be explained in terms of an imbalance between the ZHS particle size and the saturated mass of the coating material [2]. According to the results, the dispersion of ZHS particles in a polymer matrix could be increased by coating of ALP which has molar ratio of 1:1 against ZHS.

The coating technology facilitates converting the hydrophilicity of ZHS to hydrophobicity, which increases the compatibility of polymer matrix [2].

The WCA of the surface was measured to confirm the change in ZHS hydrophilic behavior after coating with ALP. As shown in Table 1, the WCA of the PANVDC decreased from 72 ± 0.3 to 36 ± 1.5 by adding ZHS. On the other hand, regarding the ALP coating on ZHS, the hydrophobicity of ZHS increased remarkably. In particular, the highest WCA was observed on the PANVDC/ALP-ZHS (1:1) composite. These results show that the ALP coating can be an effective method to reduce the water solubility of ZHS, which increases the compatibility of the composite materials and decreases the hygroscopicity of ZHS.

**Figure 2:**
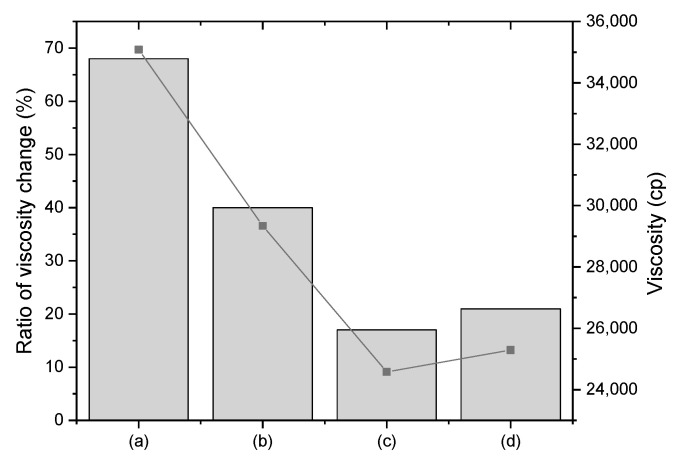
Viscosity (cP) at 100 rpm and the ratio of viscosity change (%); (**a**) PANVDC-ZHS, and PANVDC added (**b**) ALP-ZHS (1:5), (**c**) ALP-ZHS (1:1), and (**d**) ALP-ZHS (2:1) composites prepared at 50 °C for 1.5 h.

The effects of the ALP coating on the dispersion behavior of ZHS in the polymer composite were evaluated by measuring the change in polymer viscosity (cP), which was converted to the ratio of viscosity change (%). As shown in Figure 2, when ZHS alone was added to the PANVDC, the viscosity (cP) of the PANVDC increased from 20,930 cP to 35,085 cP, along with a ratio of viscosity change (%) of 68%. The change in viscosity was attributed to the addition of flame-retardant contents in the polymer composites that would lead to unacceptable values of saturation [17,18]. On the other hand, after the ALP-ZHS composite was added to PANVDC, the ratio of viscosity change (%) decreased significantly. In particular, the lowest viscosity change was obtained when ALP-ZHS (1:1) composite was added to the PANVDC. This was attributed to the improved hydrophobicity Table 1, indicating the enhanced compatibility.

A vertical flammability test (UL94) was performed to examine the effects of the ZHS and ALP-ZHS composite on the flame retardancy of the PANVDC. Table 2 lists the UL94 testing result and photographs of samples after the combustion process during 10 s. Pure PANVDC burned immediately when exposed to the flame, and extinguished itself when the flame was removed. However, it burned up to 50% of the total sample length and the remained part was also blackened by smoke occurred while burning. In case of PANVDC added ZHS, 50% of the total sample length was burned, and it presented a V-0 rating in the UL94 test. In addition, when adding ZHS-ALP composites, about 30% of the sample was burned, especially when ALP-ZHS (1:1) was added, the PANVDC film extinguished a fire as soon as it was exposed to a flame then only 15% of the total sample length was burned. Thus, according to the addition of ZHS and ALP-ZHS composites in PANVDC, not only the burning time and but also the smoke behavior decreased.

The results suggest that ALP-ZHS effects on the flame retardancy of PANVDC, which is probably due to the shielding effect of ALP and the catalyzing charring effect of ZHS [19].

Therefore, the specific molar ratio of ALP against ZHS was concluded to be 1:1, considering the results, such as the decrease in viscosity and hydrophilicity, and retention of the flame retardancy of PANVDC.

### 3.2. Characterization of the PANVDC/ALP-ZHS

**Figure 3:**
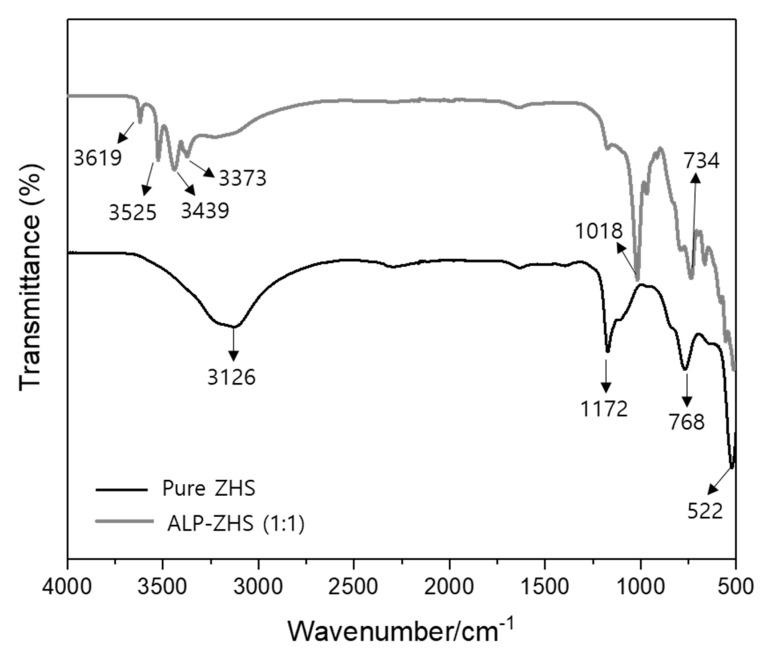
FT-IR spectra of pure ZHS, and ALP-ZHS (1:1) composite prepared at 50 °C for 1.5 h.

Figure 3 shows the FTIR spectra of the pure ZHS and ALP-ZHS composites prepared under the controlled conditions. The characteristic peaks of ZHS were centered at 3126 and 1172 cm^−1^ and were assigned to the stretching vibrations of O-H and Sn-O bonds, respectively. In addition, the peak at 768 cm^−1^ was attributed to the stretching vibrations of [Sn(OH)_6_]^2−^ belonging to ZHS [1]. After ZHS was coated with ALP, the spectra of pure ZHS was changed. The spectra of the ALP-ZHS (1:1) composite showed new absorption peaks. The band at 3619 cm^−1^ has been assigned to the surface P-OH groups, demonstrating the existence of ALP [16,20,21]. In addition, the two peaks at 1018 cm^−1^ and 734 cm^−1^ were assigned to P=O absorption bond in the phosphate and Sn-O group vibrations, respectively. Consequently, FTIR confirmed that ZHS was coated successfully with ALP, and the ALP-ZHS composite had been prepared successfully [1].

**Figure 4:**
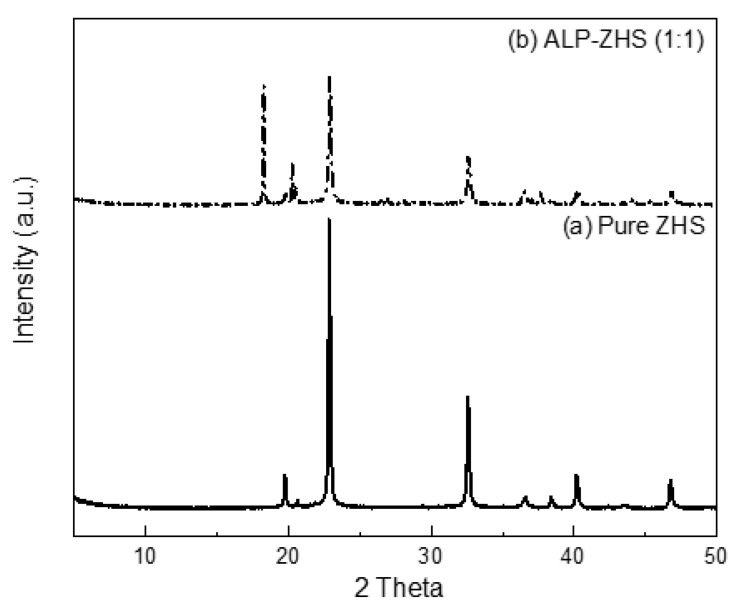
XRD patterns of (**a**) pure ZHS and (**b**) ALP-ZHS (1:1) composite prepared at 50 °C for 1.5 h.

XRD was conducted to determine the composition and crystalline phase of the as-prepared samples. Figure 4 shows the XRD patterns of pure ZHS and ALP-ZHS (1:1) composite. As shown in Figure 4a, the XRD peaks of pure ZHS were sharp, and the baselines were low. Four relatively strong peaks at 22.89°, 32.56, 40.16, and 46.83 2θ were assigned to the (200), (220), (322), and (400) reflections of the ZnSn(OH)_6_, respectively. The sharp peaks suggest that the obtained ZnSn(OH)_6_ particles have good crystallinity and no impurity phases with well-formed crystalline layered structures. For a curve of ALP-ZHS composite Figure 4b, the characteristic diffraction adsorptions of ALP were observed at approximately 18, and 21.5 2θ [22]. In the meantime, no other phases were detected by XRD, suggesting that the surface coating does not cause changes in the crystal structure of ZHS nanoparticles. Overall, ZHS was coated successfully by ALP under the controlled reaction conditions without damage to its original crystallinity structure.

**Figure 5:**
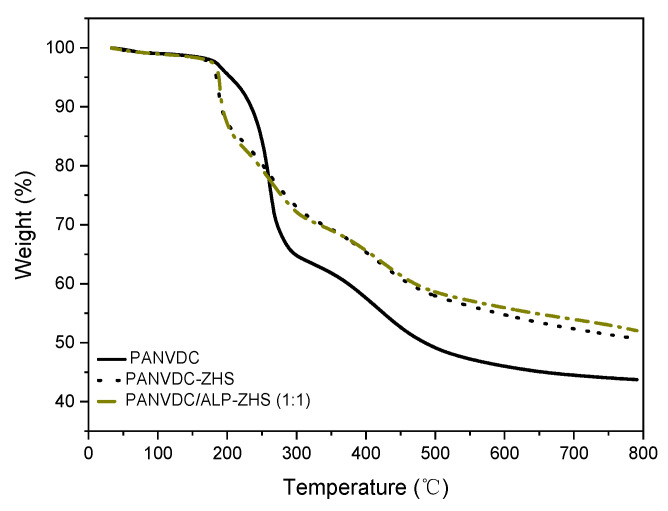
TGA curves of PANVDC, PANVDC-ZHS, and PANVDC/ALP-ZHS (1:1) films.

Figure 5 presents the TGA curve of the PANVDC, PANVDC-ZHS, and PANVDC/ALP-ZHS composites, and Table 3 lists the data. The initial decomposition temperature (T_d_) can be considered the temperature at which 5 wt. % weight loss occurs, and the temperature of the maximum mass loss rate (T_max_) is defined as the temperature at which the samples present the maximal mass loss rate. As shown in Figure 5, PANVDC showed a T_d_ at 210 °C and two main stages with T_max1_ at 302 °C and T_max2_ at 450 °C. The higher T_d_ (~210 °C) of PANVDC than PANVDC-ZHS and PANVDC/ALP-ZHS can be explained by the HCl generated through dehydrohalogenation [23]. Up to 790 °C, the residual weight of PANVDC was only 43 wt. %.

Regarding the PANVDC-ZHS and PANVDC/ALP-ZHS composites, the thermal degradation process also consisted of two main stages. From 300 °C to 450 °C, PANVDC-ZHS and PANVDC/ALP-ZHS composites decomposed slowly compared to PANVDC. This suggests that the addition ZHS enhances the thermal stability of PANVDC in a high thermal degradation process. In particular, the ALP-ZHS composite has better effects on PANVDC by increasing the pyrolysis temperature because the ALP material coated on ZHS had been exposed to the external environment and had a larger surface area, so it decomposed first [24]. In addition, active groups (-OH) were present on the surface of the ALP-ZHS composite, which resulted in the formation of a crosslinking network structure composed of flame-retardant particles and polymer chains [25].

Consequently, the maximum mass loss rate of PANVDC/ALP-ZHS composite was 54 wt. % higher than that of PANVDC and PANVDC-ZHS. TGA indicated that the addition of an ALP-ZHS composite could not change the mechanism of thermal degradation of PANVDC, but they could improve the PANVDC thermal stability of the polymer.

MCC is a useful technique for studying the relationship between the polymer chemical structure and combustion behavior based on oxygen consumption theory. Table 4 lists the relevant parameters obtained from the MCC test, such as the peak heat release rate (pHRR), time of the peak heat release rate (T_PHRR_), total heat release rate (THR), and heat release capacity (HRC).

The PANVDC film added ALP-ZHS (1:1) composites had the lowest peak of HRC, which is in good agreement with the UL94 test and pHRR value [26]. The incorporation of ZHS decreased the pHRR of PANVDC composites to 142 w/g from 167 w/g of pure PANVDC. The ALP-ZHS (1:1) composite showed a significant decrease in the pHRR value of PANVDC to 96 w/g, suggesting that the pure PANVDC burned very fast after ignition. In addition, the THR value of PANVDC decreased 13 KJ/g to 12 KJ/g, and HRC value decreased from 172 J/g·K to 114 J/g·K. These results were attributed to the shielding effect of ALP-ZHS composites, achieving the aims of synergistic flame retardancy. In addition, the barrier action of the char layer produced by the ALP-ZHS composite is expected to have important effect in hindering heat transfer and gas diffusion [27,28,29]. It is believed that the PO-radical plays the major role [29]. As a result, the charring of coated ZHS by ALP played a vital role in improving the flame retardancy behavior of PANVDC [5,17]. As a result of the LOI test, the LOI value of PANVDC increased drastically from 24 to 33% by the addition of ZHS. When ALP-ZHS (1:1) composites were added, the LOI value of PANVDC exhibited an insignificant increase while this value was still higher than the pure PANVDC. This shows that ZHS retained its original high flame retardancy, even after coating.

Therefore, the MCC and LOI test results demonstrate that the addition of ALP-ZHS (1:1) composite has a larger effect in reducing the heat release rate and total heat release and flame retardancy of PANVDC than the addition of ZHS alone.

## 4. Conclusions

This study aimed to improve the dispersion and compatibility of ZHS in a flame-retardant polymer matrix. For this, the surface of ZHS was coated by synthesizing ALP then applying the ALP-ZHS composite to the PANVDC polymer. The ALP to ZHS molar ratio was to be 1:1. The results showed that ALP-ZHS (1:1) can improve the dispersion and compatibility of ZHS in the polymer matrix and decrease the hydrophilicity and viscosity.

Moreover, a well-dispersed morphology was confirmed in the PANVDC matrix doped ALP-ZHS composite without aggregated ZHS particles. The ALP-ZHS composite had a better flame-retardant effect on PANVDC than ZHS alone. PANVDC can pass the V-0 rating in UL94, particularly the highest LOI value of 33.2% obtained when ALP-ZHS (1:1) composite was added in PANVDC.

This study examined the improvement of ZHS compatibility when added to a polymer matrix, which is a simple and effective technique for controlling ZHS as a powerful flame-retardant material that can be applied to various practical applications.

## Figures and Tables

**Table 1:** WCA of PANVDC, ZHS-PANVDC, and PANVDC films with ALP-ZHS composites prepared by different ALP molar ratios (1:5, 1:1, and 2:1) at 50 °C for 1.5 h.

Characteristics	PANVDC	PANVDC-ZHS	ALP Molar Ratio		
			1:5	1:1	2:1
WCA (°)	72 ± 0.3	36 ± 1.5	75 ± 0.8	82 ± 0.5	69 ± 1.3

**Table 2:** Results of the vertical burning test (UL94) with the photographs of PANVDC, PANVDC-ZHS, and PANVDC/ALP-ZHS composites prepared at different ALP molar ratios (1:5, 1:1, and 2:1) at 50 °C for 1.5 h.

Characteristics	PANVDC		PANVDC-ZHS		PANVDC/ALP-ZHS (1:5)		PANVDC/ALP-ZHS (1:1)		PANVDC/ALP-ZHS (2:1)		
**Burning time (sec.)**	T1	T2	T1	T2	T1	T2	T1	T2	T1	T2
	8.2 ± 0.3	4.7 ± 0.1	2.19 ± 0.2	-	0	-	0	-	0	-
**Rating**	V-2		V-0		V-0		V-0		V-0	
**UL94 test**	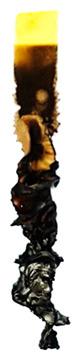		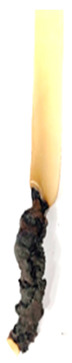		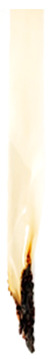		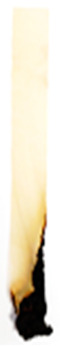		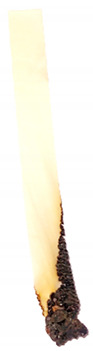		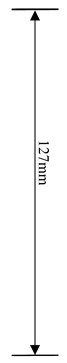

**Table 3:** TGA data of PANVDC, PANVDC-ZHS, and PANVDC/ALP-ZHS (1:1) films.

Samples	T_d_ (°C)	T_max1_ (°C)	T_max2_ (°C)	Residue (wt. %)
PANVDC	210	302	450	43
PANVDC-ZHS	190	305	505	51
PANVDC/ALP-ZHS (1:1)	190	305	510	56

**Table 4:** Results of MCC and LOI tests of pure PANVDC, ZHS-PANVDC, and PANVDC/ALP-ZHS (1:1) films.

Samples	_P_HRR (w/g)	T_PHRR_ (°C)	THR (KJ/g)	HRC (J/g·K)	LOI (%)
PANVDC	167	711	13	172	24
ZHS-PANVDC	142	684	14	149	33
PANVDC/ALP-ZHS (1:1)	96	734	12	114	33.2
